# Molecular mapping and inheritance of restoration of fertility (*Rf*) in A4 hybrid system in pigeonpea (*Cajanus cajan* (L.) Millsp.)

**DOI:** 10.1007/s00122-018-3101-y

**Published:** 2018-04-28

**Authors:** Rachit K. Saxena, Kishan Patel, C. V. Sameer Kumar, Kuldeep Tyagi, K. B. Saxena, Rajeev K. Varshney

**Affiliations:** 1 International Crops Research Institute for the Semi-Arid Tropics (ICRISAT), Hyderabad, India; 2 Krishidhan Seeds Pvt. Ltd., Hyderabad, India; 3 School of Plant Biology and Institute of Agriculture, The University of Western Australia, Crawley, WA Australia

## Abstract

**Key message:**

We report molecular mapping and inheritance of restoration of fertility (*Rf*) in A4 hybrid system in pigeonpea. We have also developed PCR-based markers amenable to low-cost genotyping to identify fertility restorer lines.

**Abstract:**

Commercial hybrids in pigeonpea are based on A4 cytoplasmic male sterility (CMS) system, and their fertility restoration is one of the key prerequisites for breeding. In this context, an effort has been made to understand the genetics and identify quantitative trait loci (QTL) associated with restoration of fertility (*Rf*). One F_2_ population was developed by crossing CMS line (ICPA 2039) with fertility restorer line (ICPL 87119). Genetic analysis has shown involvement of two dominant genes in regulation of restoration of fertility. In parallel, the genotyping-by-sequencing (GBS) approach has generated ~ 33 Gb data on the F_2_ population. GBS data have provided 2457 single nucleotide polymorphism (SNPs) segregating across the mapping population. Based on these genotyping data, a genetic map has been developed with 306 SNPs covering a total length 981.9 cM. Further QTL analysis has provided the region flanked by S8_7664779 and S8_6474381 on CcLG08 harboured major QTL explained up to 28.5% phenotypic variation. Subsequently, sequence information within the major QTLs was compared between the maintainer and the restorer lines. From this sequence information, we have developed two PCR-based markers for identification of restorer lines from non-restorer lines and validated them on parental lines of hybrids as well as on another F_2_ mapping population. The results obtained in this study are expected to enhance the efficiency of selection for the identification of restorer lines in hybrid breeding and may reduce traditional time-consuming phenotyping activities.

**Electronic supplementary material:**

The online version of this article (10.1007/s00122-018-3101-y) contains supplementary material, which is available to authorized users.

## Introduction

Pigeonpea [*Cajanus cajan* (L.) Millspaugh] is an often cross-pollinating legume crop grown in tropical and sub-tropical regions of the world. It is commonly grown in rainfed marginal lands as intercrops at subsistence level. This crop has good buffering ability against various biotic and abiotic stresses and produce reasonable amounts of yields in unfavourable conditions frequently occurring in this area makes it very useful for low-input farming systems. Despite the importance of this crop for food and nutritional security in developing countries, the yield has remained stagnant around less than one ton per hectare for almost six decades. A noticeable acceleration in the development of new cultivars of pigeonpea has been achieved during last decade with the introduction of hybrid breeding methods (Saxena et al. 2005). The hybrid seeds production is based on sporophytic cytoplasmic male sterility (CMS) system; it is also known as “three line hybrid system”. This system includes male sterile (A-line); its maintainer (B-line) and fertility restorer (R-line). A number of reports on CMS development from *Cajanus* species have been published (Ariyanayagam et al. 1995; Reddy and Faris 1981; Saxena and Kumar 2003; Wanjari and Patel 2003; Saxena et al. 2005; Mallikarjuna and Saxena 2005; Mallikarjuna et al. 2006). CMS system derived from cytoplasm of *C. cajanifolius* also known as A4 cytoplasm is now the most significant for the development of hybrid cultivars of pigeonpea (Saxena et al. 2005). The hybrids derived from A4 cytoplasm produce 30–48% more on-farm yield than the popular local varieties in multi-location field trials (Saxena and Nadarajan 2010). Although a promising technology, it often faces challenges in identifying fertility restorers, ascertaining hybrid seeds purity and maintaining the three parental (A-, B- and R-) lines. A knowledge regarding genetic control of restoration of fertility (*Rf*) will help in enhancing the efficiency of hybrid breeding. So far only limited studies have been conducted to understand the genetic systems that control *Rf* in pigeonpea (Saxena et al. 2011). The present study, therefore, was undertaken to understand the genetic systems controlling *Rf* at molecular level.

Genomics assisted breeding (GAB) has been proven a success in tackling various complex issues associated with crop improvement (Varshney et al. 2005). Genomics tools such as molecular markers and genetic maps are prerequisites for deploying any GAB programme. Genetic maps together with trait phenotyping data are required for identification of quantitative trait loci (QTLs) or genes associated with traits of interest. Advances in next-generation sequencing (NGS) and high-throughput genotyping such as genotyping-by-sequencing (GBS) have provided automation-amenable marker systems (e.g. single nucleotide polymorphism (SNP) markers) (Varshney et al. 2009). This advancement in genomics has enabled to develop rapid and high-density genetic maps in several crops including pigeonpea (Saxena et al. 2012, 2017a; b).

For strengthening the hybrid breeding, it is essential to understand the genetics and molecular mechanism involved in *Rf* and male sterility behaviour of the CMS systems. The current knowledge regarding genetics of *Rf* and its linkage with CMS, if any is not well understood in pigeonpea hybrid system. Further identification of molecular markers tightly linked to *Rf* would enhance the breeding efficiency by enabling the classification of inbred lines or germplasm as either maintainer (B-line) or restorer (R-line) without field evaluation of their test crosses; and it would also permit their rapid backcross transfer of *Rf* in elite inbred lines. A simple sequence repeat (SSR) marker-based study has reported the identification of QTLs for *Rf* in pigeonpea with large inter-marker distance in low-density genetic maps (Bohra et al. 2012). Therefore, it is highly required to develop a moderate-density genetic map that can provide tightly associated markers with *Rf.*

In view of the above in this study, we have conducted the genetic analysis, developed a relatively high-density cultivated genetic map using NGS-based GBS approach for identification of QTLs for *Rf*. Additionally PCR-based markers from the QTL region were developed for the identification of restorer lines. Subsequently PCR-based markers were validated on a set of parental lines of hybrids and segregating populations. These markers are recommended for identification of restorer lines in hybrid breeding programme.

## Materials and methods

### Mapping population and DNA extraction

One F_2_ mapping population comprising of 415 individuals derived from CMS line ICPA 2039 and its known fertility restorer ICPL 87119 was used to study the segregation pattern for *Rf*. For construction, the genetic map and QTL analysis a subset of above-mentioned population, i.e. 186 F_2_ plants was used. Genomic DNA from crossing parents and 186 F_2_ plants was isolated using NucleoSpin plant extraction kit (MACHEREY–NAGEL, USA). DNA was also isolated from parental lines (ICPA 2089, ICPA 2078, ICPA 2043, ICPA 2047, ICPA 2048, ICPA 2092, ICPB 2039, ICPB 2089, ICPB 2078, ICPB 2043, ICPB 2047, ICPB 2048, ICPB 2092, ICPR 3637, ICPR 4396, ICPR 3473, ICPR 2740, ICPR 3494, ICPR 3762) of seven hybrid combinations and another F_2_ population comprised of 75 plants derived from male sterile (KDPA514) and restorer line (KDPR857) as crossing parents used for marker validation. The quality and quantity of DNA were checked on 0.8% agarose gel.

### Phenotyping for pollen fertility

Pollen viability was used as the main criteria for assessing male fertility and sterility of individual genotypes. For pollen viability test, as described in Bohra et al. (2012), fully grown but un-opened floral buds were collected from the lower, middle and top portion of the plants between 9 am to 11 am for phenotyping the F_2_ plants. Subsequently, the mature anthers from the buds were squashed and drenched in 1% acetocarmine solution on a glass slide. Each glass slide was studied under three microscopic fields with 10× magnification. Based on microscopic view of pollens, individual plants were classified into three groups, namely as full fertile (90–100% pollen fertility), partial fertile (6–89% pollen fertility) and sterile (0–5% pollen fertility). It is also important to mention that in A4 CMS system, microsporogenesis aborts at tetrad stage where the tapetum layer persists then pollens are produced but not released (Dalvi et al. 2008). Therefore, completely sterile plants always have pollen with which to conclude 0% pollen viability. In order to study inheritance of *Rf*, phenotyping data were subjected to Chi-square analyses for testing their goodness of fit to different expected phenotypic ratios.

### Genotyping-by-sequencing (GBS) and single nucleotide polymorphism (SNP) detection

GBS approach, as mentioned in Elshire et al. (2011), was used to genotype the F_2_ mapping population (186 F_2_ plants) and parental lines. GBS libraries were prepared using *Ape*KI endonuclease. Further GBS libraries were sequenced using the Illumina HiSeq 2500 platform (Illumina Inc., San Diego, CA, USA). Sequencing data were subjected to first de-multiplexing according to the sample barcodes and adapter sequences were removed using custom pearl script. Sequencing reads were filtered following stringent criteria and reads having Phred score < 5% were discarded. Filtered sequencing reads were aligned to draft genome sequence of pigeonpea (Varshney et al. 2012) using SOAP alignments program (Li et al. 2008). Aligned sequencing reads were used for SNPs detection after quality check (Q score > 20). SNPs that were detected either heterozygous in any of the parents or present in < 50% individuals in the population were removed further and a high-quality SNP genotyping dataset was compiled.

### Genetic mapping

SNP genotyping data collected as mentioned above was used for linkage analysis using Joinmap version 4.0 (Van Ooijen and Voorrips 2006) following “Regression mapping algorithm”. Placement of markers onto different linkage groups (CcLGs) was performed using “create group using mapping tree” command. Genetic map distance was calculated using Kosambi mapping function (Kosambi 1944). Map calculations were performed with LOD value ≥ 3.0, recombination frequency ≤ 0.4 and a *χ*^2^ jump threshold for removal of loci = 5. Ripple command was used to confirm marker order in different linkage groups. Map Chart version 2.2 (Voorrips 2002) was used to construct the final genetic map. Linkage groups in genetic map were named according to pseudomolecules defined in the draft genome sequence of pigeonpea (Varshney et al. 2012).

### Quantitative trait loci (QTLs) analysis

QTL cartographer version 2.5 was used for QTLs analysis (Wang et al. 2005). QTL analysis was performed following composite interval mapping (CIM) and interval mapping (IM) approaches. CIM was performed by standard model 6 with default window size 10 cM. To obtain precise results default interval walk speed of 1 cM was selected. The forward and backward stepwise regression model was used to set number of marker cofactor as background control. The likelihood odds ratio (LOD) threshold for considering a QTL was determined by one thousand permutations. The threshold for declaring a QTL was LOD value 2.5. For each QTL, the position with the highest LOD score was considered as the most likely position of the QTL, significance level of *p* ≤ 0.05.

## Results

### Genetics of fertility restoration

A total of 15 F_1_ plants were obtained from the cross between CMS lines (ICPA 2039) and restorer line (ICPL 87119). In F_1_ generation, all 15 plants grown were male fertile, demonstrating dominance of *Rf* (Table 1). Visual observations on pollen load in the buds showed that in comparison with inbred fertile parent, i.e. ICPL 87119, the fertile hybrid plants produced almost similar quantity of pollen grains. The true hybrid nature of above-mentioned F_1_s was also assessed with SSR markers. In brief, two SSR markers, namely CcM1459 and CcM1559, were used to amplify distinct DNA fragments in ICPA 2039 (191 and 276 bp, respectively) and ICPL 87119 (187 and 272 bp, respectively) and both the alleles in F_1_s. Further a single F_1_ plant was selected based on SSR profiling and generation of maximum number of F_2_ seeds. This has provided a F_2_ population comprising 415 individual plants segregating for *Rf*. Based on the pollen fertility data, it was observed that the F_2_ population (415 plants) could be divided into three phenotypic classes (Table 1). These include fully fertile (242 plants), partial fertile (146 plants) and male sterile (27 plants). This segregation pattern had a good fit to the ratio of 9 (fully fertile): 6 (partial fertile):1 (male sterile) with probability of 0.62; these observations suggested that the full pollen fertility was conferred by two independent dominant genes with complementary gene action. The presence of at least a single dominant allele of both the genes together is essential to produce phenotypes with full pollen fertility. Further, the presence of at least one dominant allele representing either of the genes resulted in partially fertile phenotype; and all the four recessive alleles together produced male sterile plants. Therefore, expected genotypic constitutions of the observed four F_2_ plants phenotypes were *Rf1*–*Rf2*-(full fertile), *Rf1*–*rf2rf2* (partial fertile), *rf1rf1Rf2*-(partial fertile) and *rf1rf1rf2rf2* (male sterile). The male sterile phenotype expressed in only in 27 F_2_ plants, and these carried all four recessive (*rf1rf1rf2rf2*) alleles.

**Table 1:** Segregation for pollen fertility in F_2_ population derived from the cross ICPA 2039 × ICPL 87119

Genotype	Generation	Pollen set in F_2_ plants				Expected segregation ratio	*χ*^2^ value	*p* value (5%)
		Total number of plants	Number of fertile plants	Number of partial fertile	Number of sterile plants			
ICPA 2039	P_1_	10	–	–	10	–	–	–
ICPL 87119	P_2_	12	12	–	–	–	–	–
ICPA 2039 × ICPL 87119	F_1_	15	15	–	–	–	–	–
ICPA 2039 × ICPL 87119	F_2_	415	242	146	27	9:6:1	0.95	0.62

### Sequence data and SNPs discovery

In order to generate sequence data, a random subset of 186 F_2_ plants from a total of 415 F_2_ plants and parental genotypes (ICPA 2039 × ICPL 87119) were subjected to DNA isolation and library preparation. However, sequencing data could be generated on 176 F_2_ plants and parental genotypes. In total, 321.6 million reads containing ~ 33 Gb data were generated. This includes 2.7 million reads containing 6.0 Gb for ICPA 2039 and 1.8 million reads containing 4.4 Gb for ICPL 87119. In 176 F_2_ plants, the number of reads generated varied from 0.2 million (F_2__292) to 3.8 million (F_2__290) with an average of 1.8 million per F_2_ line (ESM Table 1). In order to avoid sequencing bias, F_2_ plants with less than 70 Mb data were removed from further analysis. As a result, high-quality sequencing data for 175 F_2_ plants were used for SNPs identification and subsequently parsed to remove heterozygous SNPs in parental lines. Following stringent filtering criteria mentioned in Materials and methods section, a total of 2457 SNPs were identified across 175 F_2_ plants in mapping population.

### Construction of genetic map

**Fig. 1:**
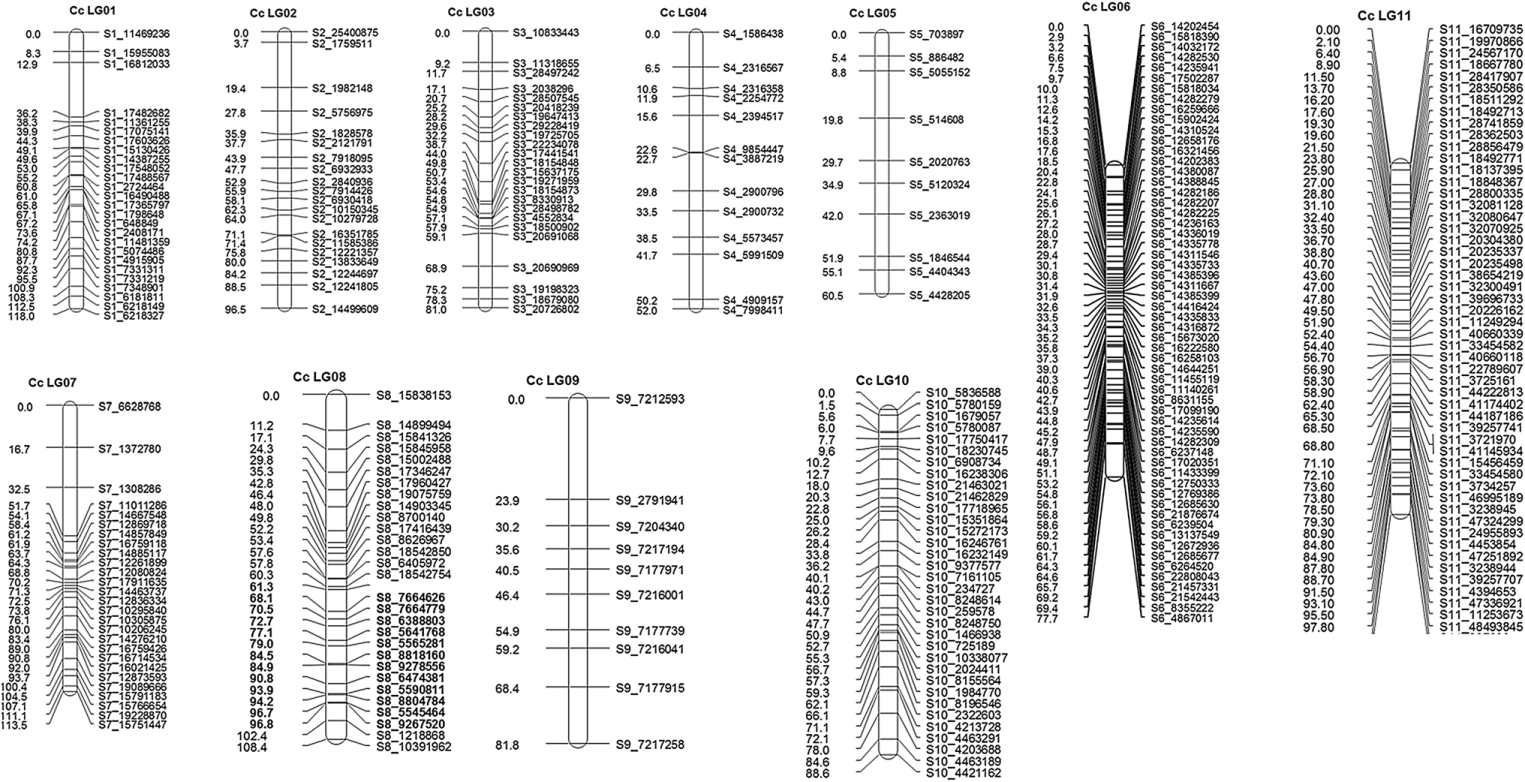
Intra-specific genetic map of pigeonpea developed on the F_2_ mapping population derived from ICPA 2039 × ICPL 87119. The map comprises 306 loci with a total length of 981.9 cM

Genotypic data for 2457 polymorphic SNPs generated on 175 F_2_ plants were used for genetic map construction. In total, 306 SNPs could be mapped on eleven linkage groups (CcLG01–CcLG11) covering 981.9 cM (Fig. 1). The highest numbers of markers were mapped on CcLG06 (58), while the lowest numbers of markers were mapped to CcLG05 (10). Length of linkage groups varied from 52 cM (CcLG04) to 118 cM (CcLG01). Highest marker density was observed for CcLG06, which had 0.8 markers per cM, whereas the lowest marker density was observed for CcLG09, which had 0.1 markers per cM. Overall, genetic map had 0.3 markers per cM (Table 2). The most spaces between two mapped SNPs were smaller than 15 cM on the CcLGs in this genetic map. However, there were six spaces where the distances between markers were large, i.e. 23.3 cM between S1_16812033 and S1_17482682 on CcLG01, 15.7 cM between S2_1759511 and S2_1982148 on CcLG02, three spaces (16.7, 15.8 and 19.2 cM between S7_6628768 and S7_1372780, S7_1372780 and S7_1308286, S7_1308286 and S7_11011286 respectively) on CcLG07 and 23.9 cM between S9_7212593 and S9_2791941 on CcLG09.

**Table 2:** Summary of the genetic map constructed using 306 SNP markers

Linkage group	Size (cM)	Number of loci	Average inter-loci distance	Number of markers per cM
CcLG01	118.0	26	4.5	0.2
CcLG02	96.5	20	4.8	0.2
CcLG03	81.0	24	3.4	0.3
CcLG04	52.0	13	4.0	0.3
CcLG05	60.5	10	6.1	0.2
CcLG06	77.6	58	1.3	0.8
CcLG07	113.5	27	4.2	0.2
CcLG08	108.4	30	3.6	0.3
CcLG09	81.8	10	8.2	0.1
CcLG10	88.5	34	2.6	0.4
CcLG11	104.1	54	1.9	0.5
Total	981.9	306	3.2	0.3

### QTL analysis for *Rf*

**Fig. 2:**
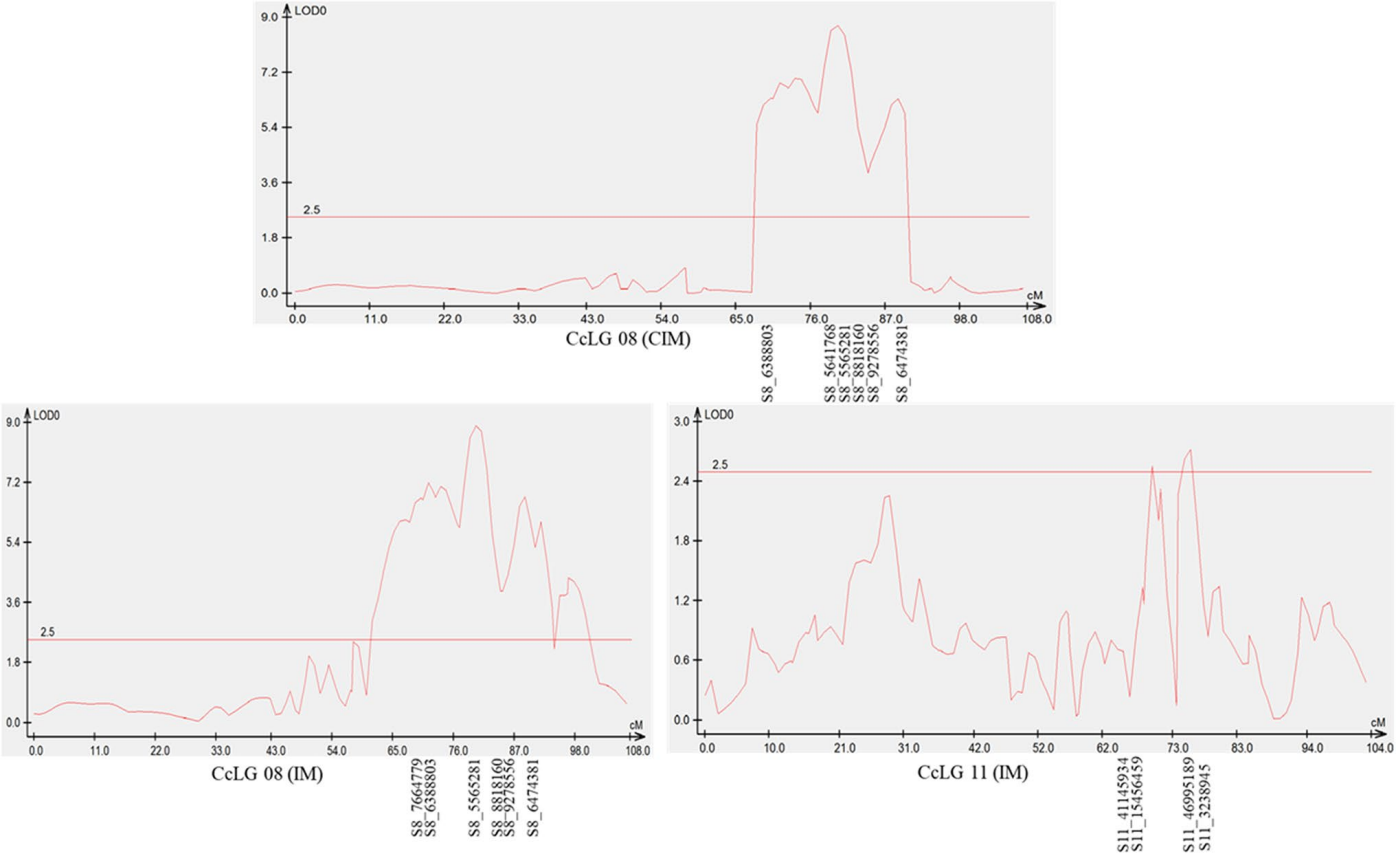
QTLs detected for restoration of fertility (*Rf*) in population derived from ICPA 2039 × ICPL 87119 using composite interval mapping (CIM) and interval mapping (IM)

QTL mapping for *Rf* was done based on mean phenotypic data of percentage pollen fertility and SNPs mapping data using two methods, namely composite interval mapping (CIM) and interval mapping (IM) (Table 3, Fig. 2). CIM analysis revealed occurrence of one major *Rf* QTL flanked by S8_6388803 and S8_6474381 markers in 72.7–90.8 cM confidence intervals on CcLG08 explaining 21.0% of phenotypic variation at 8.7 LOD value. Additionally, IM analysis has revealed two major QTLs (≥ 10% phenotypic variations), one QTL on CcLG08 and one QTL on CcLG11 and one minor QTL on CcLG11. On CcLG08 major QTL flanked by S8_7664779 and S8_6474381 markers in 70.5–90.8 cM confidence intervals explained 28.5% of phenotypic variation at 8.9 LOD value. Another major QTL detected through IM was on CcLG11 explained 11% of phenotypic variation at 2.7 LOD value flanked by S11_46995189 and S11_3238945 markers. One minor QTL was also detected on CcLG11 explained 8% of phenotypic variation at 2.6 LOD value flanked by S11_41145934 and S11_15456459 markers (Table 3, Fig. 2). On comparing the results obtained from above-mentioned two approaches, the region flanked by S8_7664779 and S8_6474381 on CcLG08 harboured major QTL and explained up to 28.5% of phenotypic variation through CIM and IM. Although a pair of QTLs with small effects (11 and 8%) and low LOD values was detected on CcLG11, they would not adequately explain the observed variation.

**Table 3:** Identification of QTLs for restoration of fertility using composite interval mapping (CIM) and interval mapping (IM)

Mapping population	Name of QTL approach	Linkage group	Confidence interval	LOD	Phenotypic variation %	Flanking markers
ICPA 2039 × ICPL 87119	CIM	CcLG08	72.7–90.8	8.7	21.0	S8_6388803 to S8_6474381
	IM	CcLG08	70.5–90.8	8.9	28.5	S8_7664779 to S8_6474381
		CcLG11	68.8–71.1	2.6	8.0	S11_41145934 to S11_15456459
		CcLG11	73.8–78.5	2.7	11.0	S11_46995189 to S11_3238945

### Development and validation of *Rf* associated markers

**Fig. 3:**
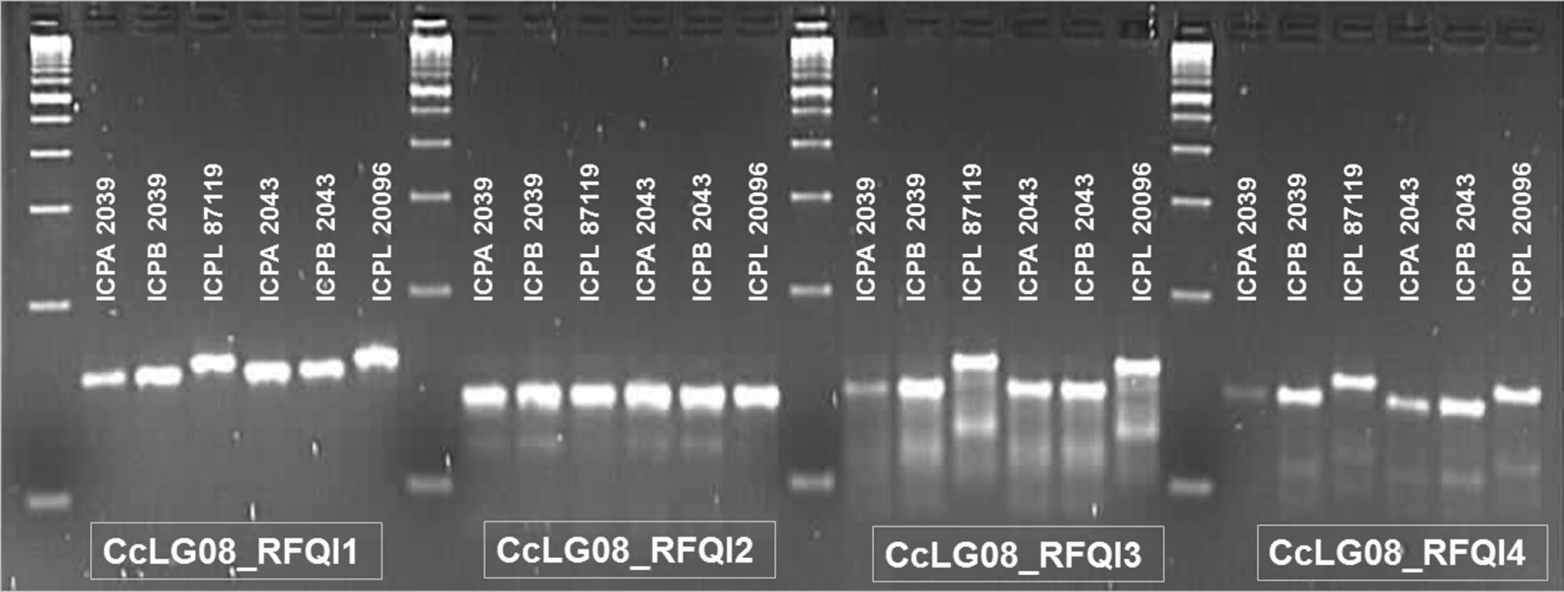
Polymorphism assessment of four Indels within the QTL region identified through sequence variations in B-lines and R-lines

In order to develop PCR-based markers associated with *Rf* in pigeonpea, we have taken advantage of available draft genome sequence information (Varshney et al. 2012) and re-sequenced data on B- and R-lines (unpublished). Sequence information within the major QTLs was extracted from the draft genome sequence of pigeonpea. Further the sequence lying under the major QTLs was compared between the two sets of maintainer and restorer lines. Re-sequence data from two maintainer lines (ICPB 2039 and ICPB 2043) constituted a “B-set” and from three restorer lines (ICPL 20097, ICPL 20098 and ICPL 8705) constituted an “R-set”. Sequence comparisons between “B-set” and “R-set” have provided a total of 259 sequence variations in form of SNPs and Indels (ESM Table 2). Further a stringent filtering criteria was used and only homozygous calls (within the particular set) and contrasting variations between “B-set” and “R-set” were retained. As a result, a total of 185 sequence variations (172 SNPs and 13 Indels) identified in QTL region between “B-set” and “R-set”. One of the primary objectives of this analysis was the development of PCR-based marker which could be amenable for low-cost genotyping. As for SNPs genotyping sophisticated assays are required and generally not feasible to every laboratory. Therefore, we have targeted Indels with reasonable nucleotide differences between “B-set” and “R-set”. Based on the length variation out of 13 Indels four were selected for primer pairs designing (ESM Table 3). Subsequently, primer pairs were used to amplify target Indels and checking their ability to differentiate “*rf allele*” containing non-restorer lines, i.e. A-lines, B-lines with the “*Rf allele*” containing restorer (R-) lines in two sets on 3.5% agarose gel. Out of four targeted Indels three Indels could differentiate “*rf allele*” containing non-restorer lines with the “*Rf allele*” containing restorer lines. One Indel (CcLG08_RFQI2)-based primer pair produced monomorphic band in all the tested lines (Fig. 3).

Assessment of the utility of PCR-based markers from the QTL region in terms of differentiating non-restorer lines and restorer lines was further performed on seven sets of parental lines used for hybrid production in pigeonpea. This panel of lines also included two sets of parental lines used for above-mentioned primary assessment of Indel polymorphism on agarose gel. Based on the agarose gel profiles two Indels, i.e. CcLG08_RFQI1 and CcLG08_RFQI4 were used for further assessment, whereas, one Indel CcLG08_RFQI3 left out due to multiple bands.

**Fig. 4:**
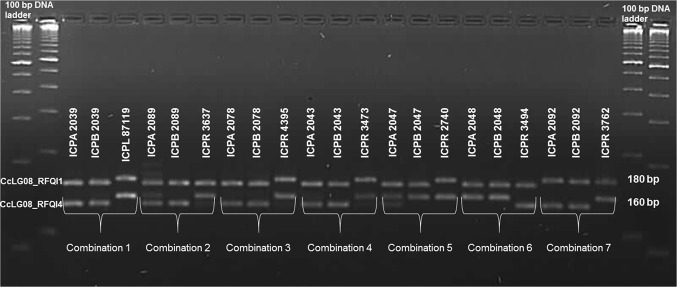
Validation of CcLG08_RFQI1 and CcLG08_RFQI4 markers in separating restorer lines from the non-restorer (A- and B-) lines in seven different combinations. Amplicons of the same line obtained using CcLG08_RFQI4 (~ 160 bp) and CcLG08_RFQI1 (~ 180 bp) markers were loaded in the same well at 20-min time interval

The marker CcLG08_RFQI1 could differentiate restorer lines from non-restorer lines in four out of seven tested combinations. While using CcLG08_RFQI4 marker, six out of seven combinations could be differentiated into restorer lines and non-restorer lines. It was predicted that simultaneous application of a combination of markers should increase the efficiency of selection. However, when both markers were analysed together, all non-restorer lines were separated from restorer lines in seven combinations (Fig. 4).

Further to generate single gel-based profiles for both markers three different experiments were conducted: (1) amplified products from both the markers loaded in same well at 20-min interval, i.e. amplicon of the same line obtained by using CcLG08_RFQI4 (~ 160 bp) marker was loaded in gel and run for 20 min, and subsequently amplicon for the same line obtained by using CcLG08_RFQI1(~ 180 bp) marker was loaded in the same well (Fig. 4), (2) amplicons for the same line obtained by using CcLG08_RFQI4 and CcLG08_RFQI1 markers were loaded in the same well (ESM Fig. 1), and (3) single PCR was set up with primer pairs for both CcLG08_RFQI4 and CcLG08_RFQI1 markers and loaded on agarose gel (ESM Fig. 2). Clear profile for each line with both the markers was seen in the first case where separately amplified PCR product was loaded in the same well but after 20-min time interval. In the remaining two cases, convincing line profiles could not be obtained, this may require further optimization to standardize mixed loading or amplification in a single reaction.

### Segregation of *Rf* associated markers

The primer pairs designed for CcLG08_RFQI1 and CcLG08_RFQI4 were used to genotype F_2_ population derived from cross ICPA 2039 × ICPL 87119 (ESM Figs. 3, 4). Both markers correlated with the pollen viability test for fertility and sterility and differentiated all 188 F_2_ plants into fertile plants and sterile plants (CcLG08_RFQI1: 148 fertile and 36 sterile, no amplification: 4; CcLG08_RFQI4: 147 fertile and 36 sterile, no amplification: 5). A goodness-of-fit test for a 1 sterile (homozygous): 2 fertile (heterozygous):1 fertile (homozygous) provided a *χ*^2^ value of 7.8, 10.1 at probability (*p*) value of 0.02, 0.01 for CcLG08_RFQI1 and CcLG08_RFQI4, respectively. Further to validate these two markers in another genetic background, F_2_ population comprised of 75 individuals derived from male sterile (KDPA514) and restorer line (KDPR857) as crossing parents were used. Interestingly CcLG08_RFQI1 segregated in this population with a *p* value 0.57 and could differentiate male sterile (22 F_2_ plants), male fertile (18 F_2_ plants), heterozygote (33F_2_ plants) and no amplification identified in two F_2_ individuals (ESM Fig. 5). However, CcLG08_RFQI4 did not segregate in this population. Above-mentioned validation experiments (testing in parental lines of hybrids and F_2_ populations) clearly demonstrated that both makers should be used in combination for the identification of *Rf* allele containing lines in a breeding programme.

## Discussion

Like many other crops, CMS in pigeonpea is a maternally inherited trait and associated with certain unusual open reading frames in mitochondrial genome (Tuteja et al. 2013). The *Rf* genes present in nucleus suppress the male sterility and allows the production of viable pollen grains. The level of *Rf* varies in different hybrid systems and depends on number of *Rf* genes present in restorer lines (Saxena et al. 2014). It has also seen that environmental factors may play role in the expression of pollen fertility (Kaul 1988). Therefore, it is essential to identify restorer lines which have all *Rf* genes and provide full pollen load in hybrids to produce high yields. To achieve this, understanding inheritance of *Rf* is essential. It is important to mention that pigeonpea is the only legume crop where CMS-based hybrid system has been used for commercial purpose. This hybrid system is based on A4 cytoplasm (Saxena et al. 2005). In the present study, F_1_ and F_2_ generations were developed to determine the nature of gene action and segregation pattern of *Rf* genes in A4 CMS system. Two independent dominant *Rf* genes with complementary action were identified in the tested population. The present study supports previous findings in pigeonpea where two dominant genes were found responsible for *Rf* in a number of segregating populations (Saxena et al. 2011). In few cases, single dominant gene was also found responsible for *Rf* (Saxena et al. 2011). Further, restorer lines carrying two dominant genes were found more stable across the locations as compared to restorer lines carrying one dominant gene in terms of their capacity of restoring fertility in hybrids (Saxena et al. 2011). Therefore, it is useful to map the *Rf* locus in pigeonpea and develop user friendly markers to differentiate restorer lines from non-restorer lines.

The first-generation genetic maps of pigeonpea were based on inter-specific mapping population. One of the very first such genetic map developed in pigeonpea was based on diversity array technology (DArT) markers (Yang et al. 2011). Subsequently SSR marker-based genetic map was developed by Bohra et al. (2011). Soon after this, intra-specific genetic maps were also generated using SSR markers with relatively very low marker density (Gnanesh et al. 2011; Bohra et al. 2012). SNP-based genotyping assays were also deployed for genetic map construction using inter-specific (Saxena et al. 2012) and intra-specific mapping populations (Kumawat et al. 2012).

NGS coupled with available draft genome sequences in many crop species have provided an opportunity to deploy advanced methodologies for SNP discovery and genotyping. One of such NGS-based technology is GBS. GBS has been used for the highly efficient and cost-effective discovery of SNPs in different species (Kumar et al. 2012, Jaganathan et al. 2015) including pigeonpea (Saxena et al. 2017a;b). Generally, GBS provides higher numbers of relatively evenly distributed sequence variations (SNPs) as compared to previous methods used for variations detection. GBS also provides single step process for SNP discovery and genotyping which directly can be used for different studies such as assessment of genetic diversity, construction of genetic maps and detection of markers linked with important traits. Therefore, GBS was used for developing an intra-specific genetic map of the pigeonpea genome in the present study. Implementation of GBS has resulted in a significant enhancement of marker density (0.3 markers/cM) in intra-specific genetic map for *Rf*.

Previously published results of mapping the restoration of fertility in A4 cytoplasm were achieved with the use of three F_2_ mapping populations comprising of 188 individuals each (Bohra et al. 2012). A similar number of F_2_ individuals were used for the development of GBS-based genetic map in the present study. However, the present genetic map has almost two to three folds’ higher numbers of markers mapped (306 SNPs) as compared to any of the previous three genetic maps (78-140 SSRs). In the previous studies, a total of four major QTLs were detected in SSR-based study and a common QTL was found in two mapping populations. The majority of the QTLs identified were located on the LG06 in all the three mapping populations. In order to compare QTLs detected in Bohra et al. (2012) with the present findings, we have extracted bacterial artificial chromosome (BAC)-end sequences for corresponding SSRs and mapped to present GBS data. Interestingly majority of the major QTLs detected in Bohra et al. (2012) were collocated with the present study on CcLG08 (ESM Table 4). Comparative analysis between above-mentioned studies has further enhanced our confidence in this region at CcLG08. Further, this region was used to develop user friendly PCR-based markers. PCR-based markers localized in this target region for the selection of restorer lines are very useful for practical applications. In the present study, two informative markers were developed. Both markers showed statistically significant results; however, the precision of selection by using single marker was not perfect. The application of both markers in combination reached up to 100% perfection in discriminating restorer lines from non-restorer lines. Therefore, the efficiency of selection for *Rf* will be enhanced with the application of both markers in combination.

## Conclusion

The genetic map of pigeonpea with major QTL on CcLG08 reported here also confirms the efficiency of NGS-based GBS technology. To our knowledge, this is the first report on the application of GBS for mapping *Rf* in pigeonpea, even if resolution of the genetic map is at moderate level and not very high. The results of QTL mapping indicates that the genomic region identified on CcLG08 restores male fertility in pigeonpea. Further use of genome sequence information in conjunction with QTL mapping by GBS method has provided an alternate approach for mapping and rapid development of user friendly markers. This approach has provided almost all possible sequence variations in the target region and will be useful in future fine mapping efforts. On the other hand, developed PCR-based markers will reduce traditional labour-intensive and time-consuming phenotyping activities for the identification of restorer lines. Moreover, developed PCR-based markers in this study are amenable to low-cost genotyping and can be performed in any laboratory with basic facilities of PCR and electrophoresis. This ease in marker genotyping will certainly help to overcome the technical expertise required for advanced genotyping methodologies.

### Author contribution statement

RKS and RKV conceived, designed and coordinated the experiments. RKS, KP, KT, CVSK and KBS performed the field experimentations. RKS and KP performed the genotyping. RKS, KP, CVSK, KBS and RKV analysed the data. RKS, RKV and KBS interpreted results. RKS, KBS and RKV wrote the paper.

## Electronic supplementary material

Below is the link to the electronic supplementary material.

**ESM Fig.** **1** Amplified products from CcLG08_RFQI4 and CcLG08_RFQI1 were mixed in equal concentrations and loaded in single well (PPTX 169 kb)

**ESM Fig.** **2** Amplified product obtained through single PCR using CcLG08_RFQI4 and CcLG08_RFQI1 primer pairs and loaded in agarose gel (PPTX 177 kb)

**ESM Fig.** **3** Segregation pattern CcLG08_RFQI1 in F_2_ population derived from ICPA 2039 × ICPL 87119 (PPTX 132 kb)

**ESM Fig.** **4** Segregation pattern CcLG08_RFQI4 in F_2_ population derived from ICPA 2039 × ICPL 87119 (PPTX 241 kb)

**ESM Fig.** **5** Segregation pattern CcLG08_RFQI1 in second F_2_ population derived from different set of male sterile (KDPA514) and restorer line (KDPR857) (PPTX 87 kb)

**ESM Table** **1** Sequencing data generated on parental lines and F_2_ plants (XLSX 15 kb)

**ESM Table** **2** Sequence variations detected in the QTL region (XLSX 37 kb)

**ESM Table** **3** Details on four target Indels identified in the QTL region and primer sequences (XLSX 9 kb)

**ESM Table** **4** A comparative analysis between SSR-based study (Bohra et al. 2012) and SNP-based present study (XLSX 11 kb)
