# Supplementary material for: Molecular mapping and inheritance of restoration of fertility (Rf) in A4 hybrid system in pigeonpea (Cajanus cajan (L.) Millsp.)
Source: Theor Appl Genet. 2018 Apr 28;131(8):1605–14. doi: 10.1007/s00122-018-3101-y (PMC6061154; doi:10.1007/s00122-018-3101-y)
Supplement: Supplementary file 1 — ESM Fig. 1 Amplified products from CcLG08_RFQI4 and CcLG08_RFQI1 were mixed in equal concentrations and loaded in single well (PPTX 169 kb) [file 122_2018_3101_MOESM1_ESM.pptx]

## Slide 1
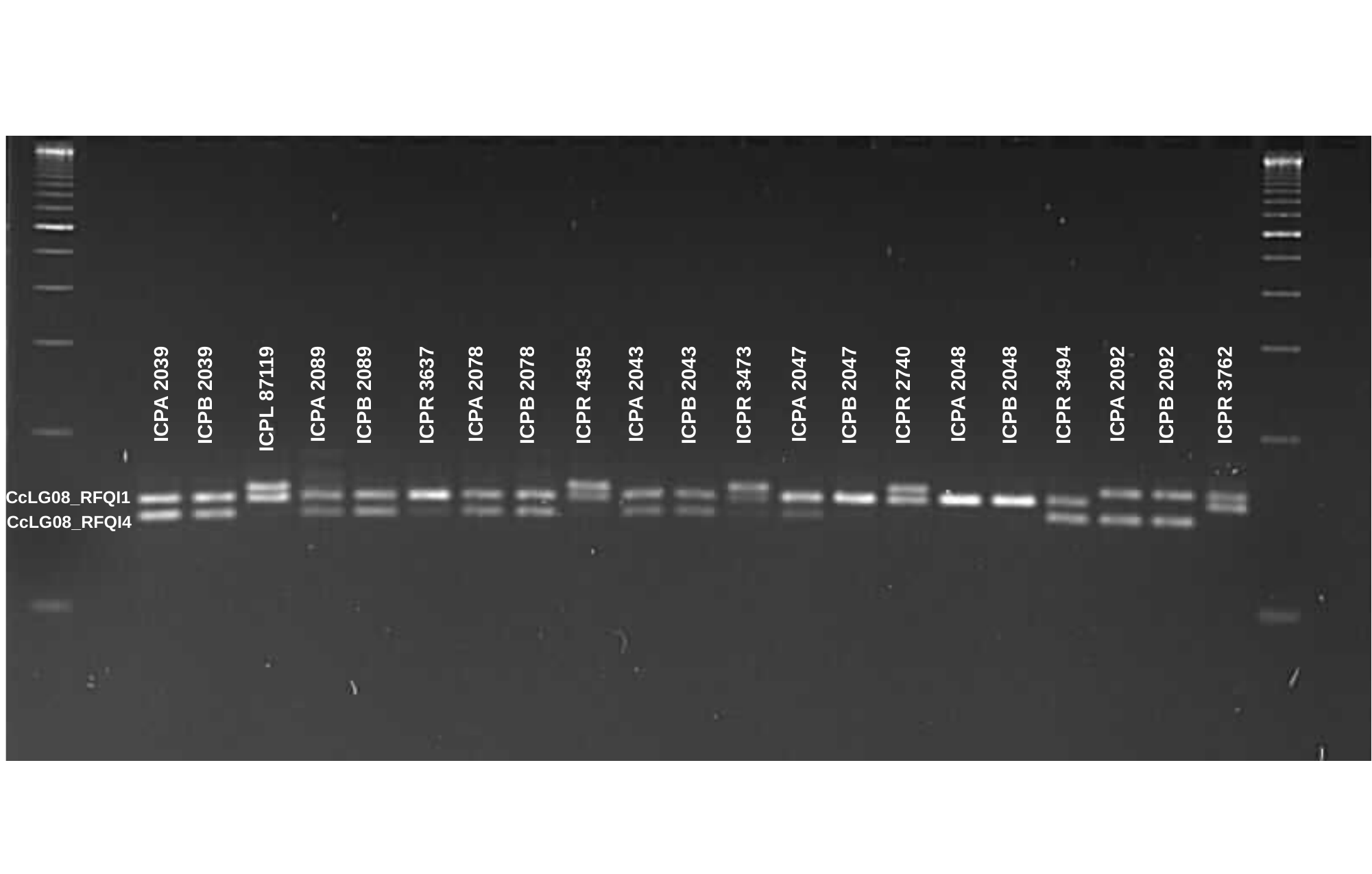

ICPA 2039
ICPA 2089
ICPA 2078
ICPA 2043
ICPA 2047
ICPA 2048
ICPA 2092
ICPB 2039
ICPB 2078
ICPR 2740
ICPB 2048
ICPR 3494
ICPB 2092
ICPR 3762
ICPB 2089
ICPR 3637
ICPR 4395
ICPB 2043
ICPR 3473
ICPB 2047
ICPL 87119
CcLG08_RFQI1
CcLG08_RFQI4
