# Supplementary material for: Molecular mapping and inheritance of restoration of fertility (Rf) in A4 hybrid system in pigeonpea (Cajanus cajan (L.) Millsp.)
Source: Theor Appl Genet. 2018 Apr 28;131(8):1605–14. doi: 10.1007/s00122-018-3101-y (PMC6061154; doi:10.1007/s00122-018-3101-y)
Supplement: Supplementary file 4 — ESM Fig. 4 Segregation pattern CcLG08_RFQI4 in F2 population derived from ICPA 2039 × ICPL 87119 (PPTX 241 kb) [file 122_2018_3101_MOESM4_ESM.pptx]

## Slide 1
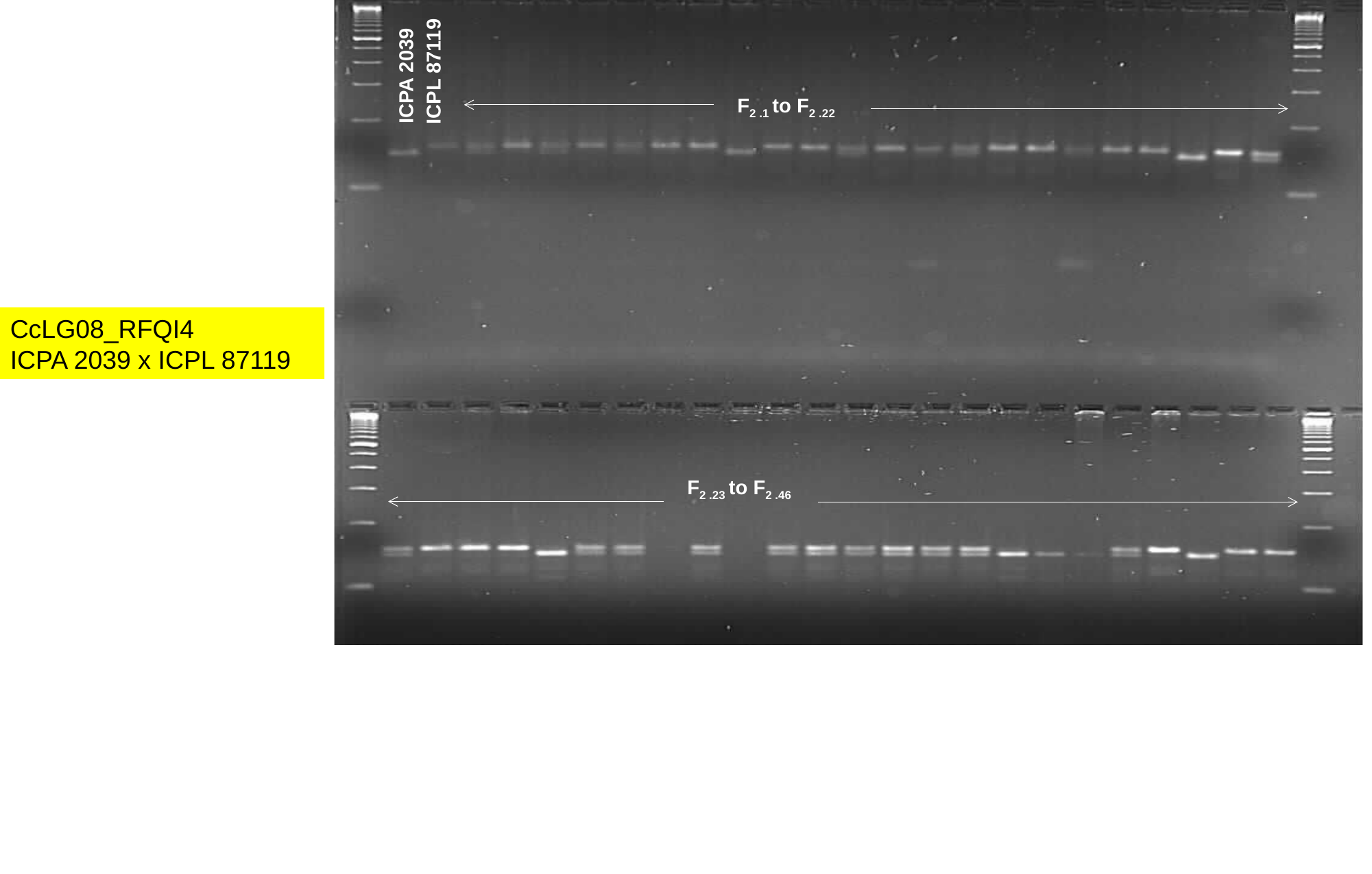

ICPL 87119
ICPA 2039
 F2 .1 to F2 .22
CcLG08_RFQI4
ICPA 2039 x ICPL 87119
 F2 .23 to F2 .46
 F2 .23 to F2 .46

## Slide 2
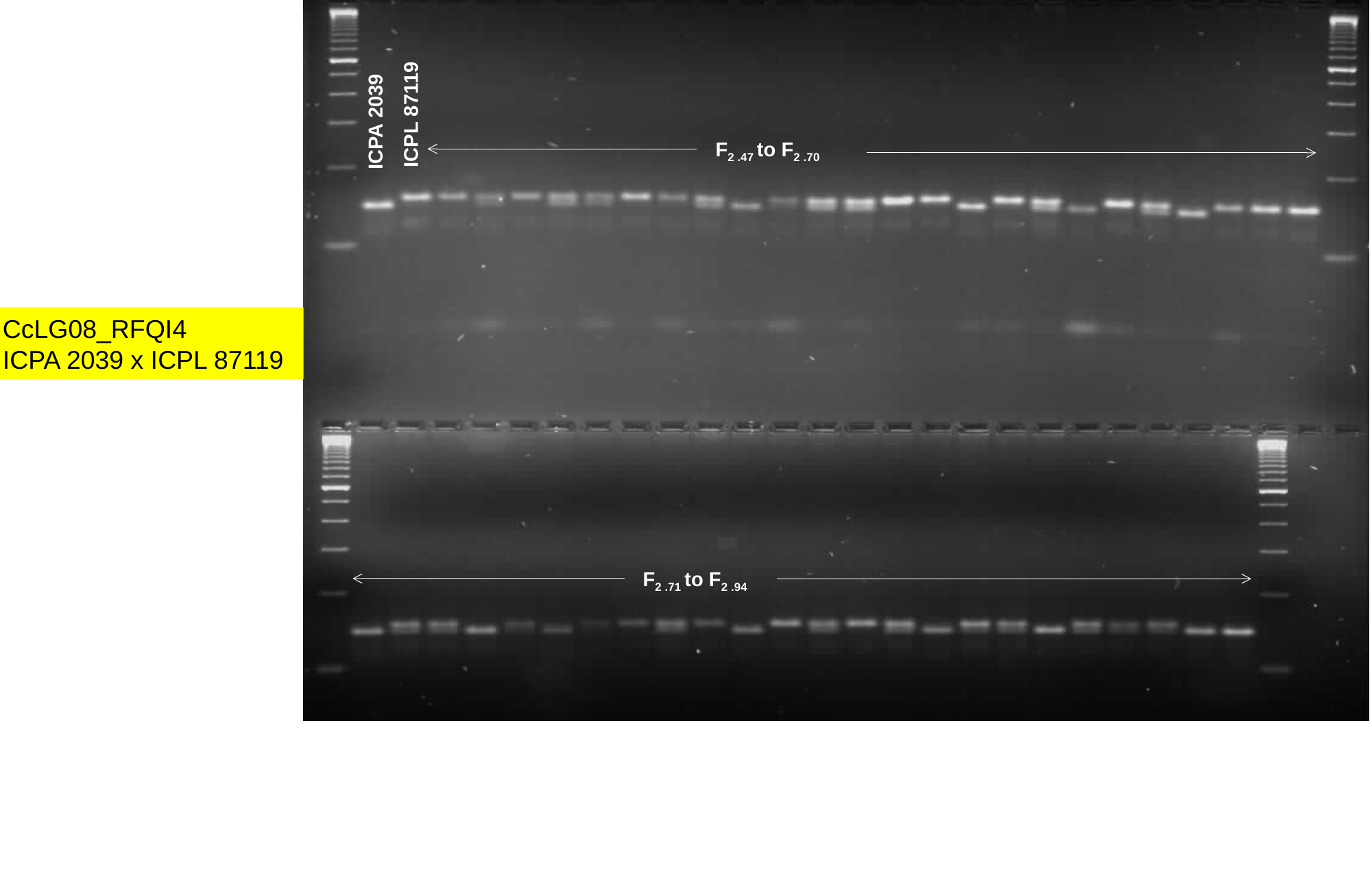

ICPL 87119
ICPA 2039
 F2 .47 to F2 .70
CcLG08_RFQI4
ICPA 2039 x ICPL 87119
 F2 .71 to F2 .94

## Slide 3
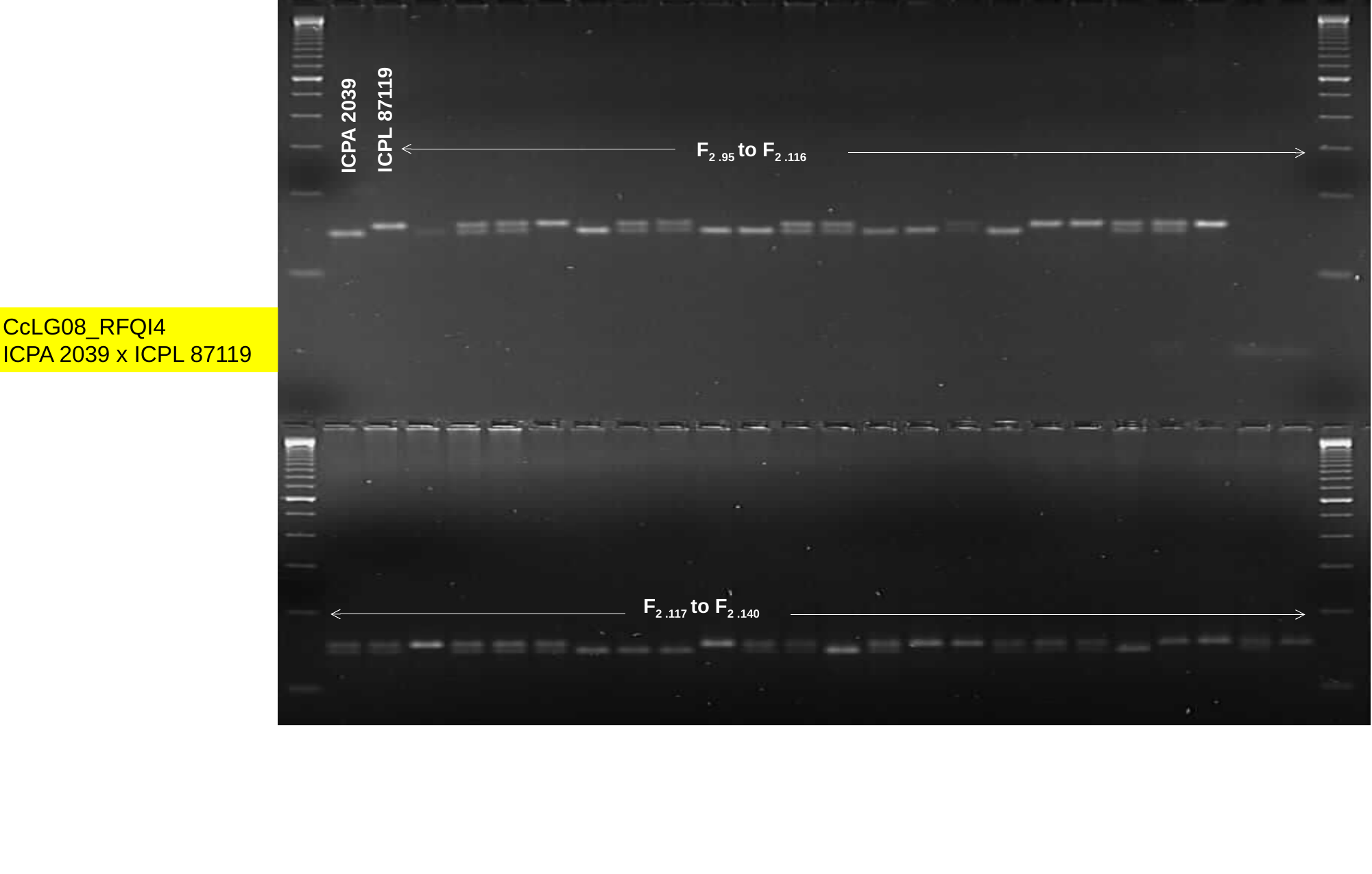

ICPL 87119
ICPA 2039
 F2 .95 to F2 .116
CcLG08_RFQI4
ICPA 2039 x ICPL 87119
 F2 .117 to F2 .140

## Slide 4
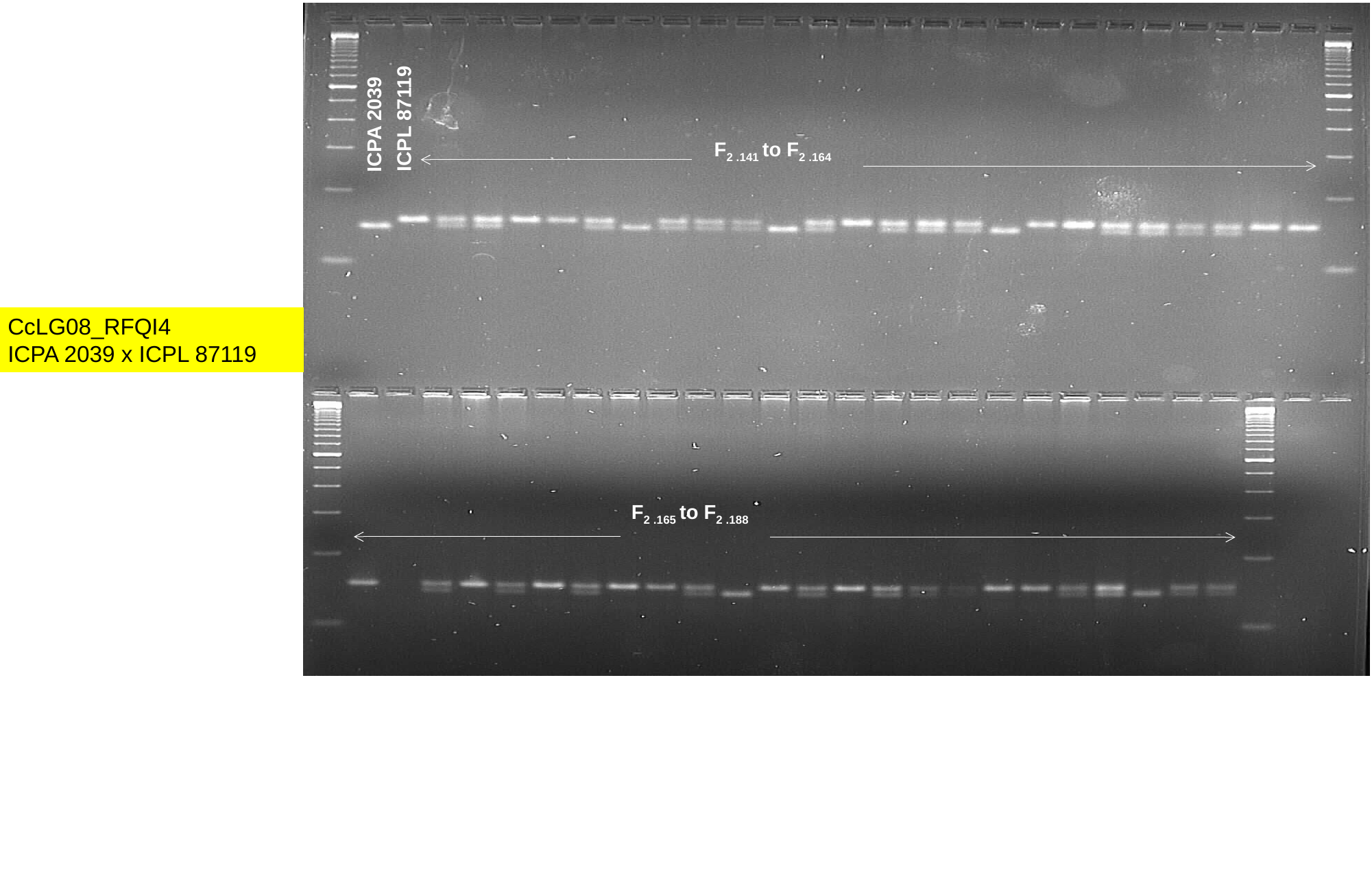

ICPL 87119
ICPA 2039
 F2 .141 to F2 .164
CcLG08_RFQI4
ICPA 2039 x ICPL 87119
 F2 .165 to F2 .188
