# Supplementary figures and images for: Molecular mapping and inheritance of restoration of fertility (Rf) in A4 hybrid system in pigeonpea (Cajanus cajan (L.) Millsp.)
Source: Theor Appl Genet. 2018 Apr 28;131(8):1605–14. doi: 10.1007/s00122-018-3101-y (PMC6061154; doi:10.1007/s00122-018-3101-y)

## Slide 1
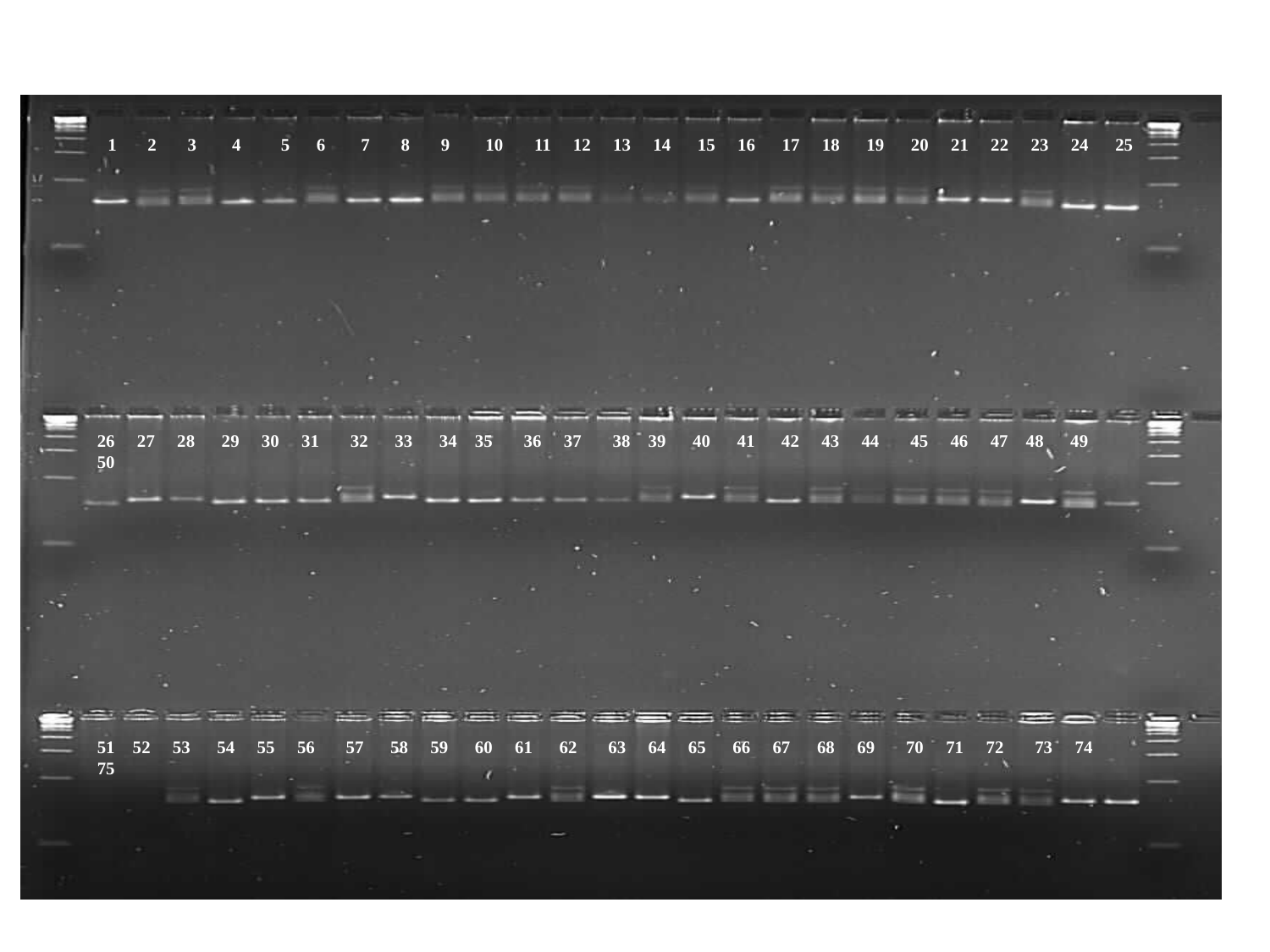

1 2 3 4 5 6 7 8 9 10 11 12 13 14 15 16 17 18 19 20 21 22 23 24 25
26 27 28 29 30 31 32 33 34 35 36 37 38 39 40 41 42 43 44 45 46 47 48 49 50
51 52 53 54 55 56 57 58 59 60 61 62 63 64 65 66 67 68 69 70 71 72 73 74 75

Supplement: Supplementary file 5 — ESM Fig. 5 Segregation pattern CcLG08_RFQI1 in second F2 population derived from different set of male sterile (KDPA514) and restorer line (KDPR857) (PPTX 87 kb) [file 122_2018_3101_MOESM5_ESM.pptx]
